# Translational Approach to Behavioral Learning: Lessons from Cerebellar Plasticity

**DOI:** 10.1155/2013/853654

**Published:** 2013-11-11

**Authors:** Guy Cheron, Bernard Dan, Javier Márquez-Ruiz

**Affiliations:** ^1^Laboratory of Electrophysiology, Université de Mons, 7000 Mons, Belgium; ^2^Laboratory of Neurophysiology and Movement Biomechanics, CP640, ULB Neuroscience Institut, Université Libre de Bruxelles, 1070 Brussels, Belgium; ^3^Department of Neurology, Hôpital Universitaire des Enfants Reine Fabiola, Université Libre de Bruxelles, 1020 Brussels, Belgium; ^4^División de Neurociencias, Universidad Pablo de Olavide, 41013 Sevilla, Spain

## Abstract

The role of cerebellar plasticity has been increasingly recognized in learning. The privileged relationship between the cerebellum and the inferior olive offers an ideal circuit for attempting to integrate the numerous evidences of neuronal plasticity into a translational perspective. The high learning capacity of the Purkinje cells specifically controlled by the climbing fiber represents a major element within the feed-forward and feedback loops of the cerebellar cortex. Reciprocally connected with the basal ganglia and multimodal cerebral domains, this cerebellar network may realize fundamental functions in a wide range of behaviors. This review will outline the current understanding of three main experimental paradigms largely used for revealing cerebellar functions in behavioral learning: (1) the vestibuloocular reflex and smooth pursuit control, (2) the eyeblink conditioning, and (3) the sensory envelope plasticity. For each of these experimental conditions, we have critically revisited the chain of causalities linking together neural circuits, neural signals, and plasticity mechanisms, giving preference to behaving or alert animal physiology. Namely, recent experimental approaches mixing neural units and local field potentials recordings have demonstrated a spike timing dependent plasticity by which the cerebellum remains at a strategic crossroad for deciphering fundamental and translational mechanisms from cellular to network levels.

## 1. Introduction

**Figure 1:**
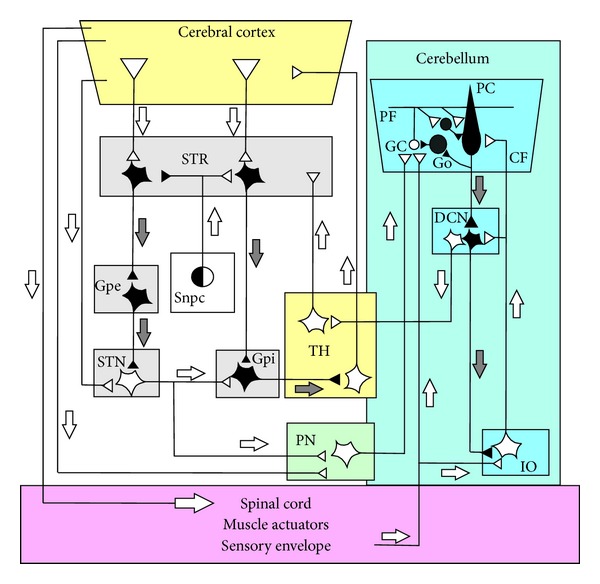
Schematic diagram of the circuits interconnecting the olivocerebellum, the thalamus, the basal ganglia, the pontine nuclei, the cerebral cortex, and the spinal cord. The part of the circuit showing anatomical links between the basal-ganglia and the cerebellum is adapted from recent anatomical experiments using retrograde transneuronal transport of rabies virus from injections into the cerebellar cortex and in nuclei of basal ganglia, establishing evidence for disynaptic pathways that directly link the cerebellum with the basal ganglia (see [1] for a review). Injection of the rabies virus into the cerebellar cortex induced two stages of transport: retrograde transport to first-order neurons in the pontine nuclei (PN) that innervate the injection site and then retrograde transneuronal transport to second-order neurons in the subthalamic nucleus (STN) that innervate the first-order neurons [28]. Injection of rabies virus into the striatum (STR) induced retrograde transport to first-order neurons in the thalamus (TH) that innervate the injection site and then retrograde transneuronal transport to second-order neurons in the dentate nucleus (DN) that innervate the first-order neurons [29]. In addition, the striatal neurons that receive cerebellar inputs include neurons in the “indirect” pathway that send projections to the external globus pallidus (Gpe). The classical network of the basal ganglia (*adapted from *[30]) represented by the parallel “direct” and “indirect” pathways from the STR to the basal output nuclei. The “direct” path sent inhibitory input from the SRT to the internal part of the globus pallidus (Gpi). The basal ganglia circuit is completed by the dopaminergic pathway and represented the projection of the substantia nigra pars compacta (Snpc) to the STR. Inhibitory neurons are shown as filled symbols, excitatory neurons by open symbols. Grey and white arrows represent inhibitory or excitatory pathways, respectively. Abbreviations: PC, Purkinje cell; GO, Golgi cell; DCN, deep cerebellar nuclei; PF, parallel fiber; GC granule cell; IO, inferior olive; CF, climbing fiber.

Recent evidences show that the cerebellum plays a key role in motor and nonmotor domains through a great number of cerebro-cerebellar closed loops [1] (Figure 1) that sustain different forms of learning [2–9]. In this context, it is widely admitted that synaptic plasticity underlies learning and memory [10–15] and that the Purkinje cell (PC), which is the sole output neuron of the cerebellar cortex, can learn up to 5 kilobytes of information corresponding to 40,000 input-output associations [16]. This high learning capacity of the PC promotes this type of neuron at the first place for revisiting the different approaches already performed in studying plasticity in cerebellum.

The crucial position of the cerebellum in brain circuitry and its involvement in sensorimotor and cognitive processing make it an ideal structure for studying the possible role of neuronal plasticity in a translational perspective on learning. The attractiveness of the cerebellum in the context of learning results is enhanced because it can be modeled as a neuronal network in terms of a “wiring diagram” [17], in which input-output signaling can be identified [18–25]. The PC can thus be seen as a microcosm within the cerebellum with multiple short-scale feed-forward and feedback loops inside of the cerebellar cortex itself (see [26] for a review). This contributes to the extreme complexity that makes accurate determination of their final function and their implication in learning and memory difficult.

In order to establish a comprehensive theory of learning, we need to determine a chain of causalities linking together neural signals, plasticity mechanisms, neural circuits, and behavioral learning [27]. To usefully test hypotheses within the framework of such a theory, this chain of interactions needs to be studied in the physiology of behaving or alert animals. Here, we critically review this translational approach in three well-characterized forms of learning: (1) the adaptation of the vestibuloocular reflex (VOR) and the smooth pursuit control, (2) the classical conditioning of the eyeblink, and (3) the plasticity of the sensory cutaneous envelope.

## 2. Cerebellar Cortical State Determined by Purkinje Cell Signaling

Whatever the type of the involved paradigms, the output of the cerebellar cortex is always determined by the PC firing. This major signaling can be electrophysiologically identified by two types of firing patterns, namely, complex spikes (CS) [31] and simple spikes (SS). CS are fired in response to climbing fiber (CF) activity in an all-or-none mode of production [32, 33]. They are characterized by a first fast sodium spike followed by a high-frequency burst of spikelets and occur at a low frequency rate around 1 Hz [34] but can reach higher frequencies, for example, during nociceptive stimulation (~11 Hz, [35]) or learning (~5 Hz, [36, 37]). CFs are also able to impose a rhythmic template throughout the PC population [38–40]. In addition, CF activity produces large, widespread calcium transients in PC dendrites [41]. This has been proposed to be a prerequisite for the induction of long-term depression (LTD) [42]. 

SS firing presents a resting state at about 50 Hz in alert animal [34, 43–48]. It results from the conjugated action of (1) excitatory postsynaptic potential (EPSP) produced by the synapse between the ascending portion of a granular cell (GC) axon and a PC dendrite [49, 50], (2) graded but less efficient EPSP from parallel fibers (PFs) [51], (3) strong modulation by the CF [44, 52, 53], (4) inhibitory postsynaptic potential (IPSP) from the inhibitory interneurons such as stellate, basket, and Lugaro cells [54], and (5) intrinsic pacemaker activity [55–58]. 

The specific discharge frequencies of CS and SS are reciprocally organized, that is, an increase in CS is accompanied with a decrease in SS, and *vice versa* [59–62]. This has been suggested to be essential for motor coordination. Recent experimental results support this suggestion. Ptf1a::cre;Robo3lox/lox mice showed a selective CF rerouting from a contralateral to an ipsilateral projection [63]. Three main effects are produced by this CF rerouting experiment in addition to reversion of CS modulation: (1) it also produces reversion of SS modulation, (2) it converts the phase of the inhibitory interneurons, and (3) it produces severe ataxia.

## 3. The VOR and Smooth Pursuit Control: The First Cerebellar Lesson

Since Ito's original proposal [64] that the cerebellar flocculus was implicated in the calibration of the VOR, the adaptation of this reflex unambiguously linked to cerebellar function [65] *in fine* implies one site of plasticity in the cerebellum and another one in the brainstem. This conclusion has been accepted following a protracted controversy between cerebellum [66] and brainstem [67] “supporters” and has now opened an ideal field for the study of the bidirectional dialogue between the brainstem and cerebellum circuits. 

**Figure 2:**
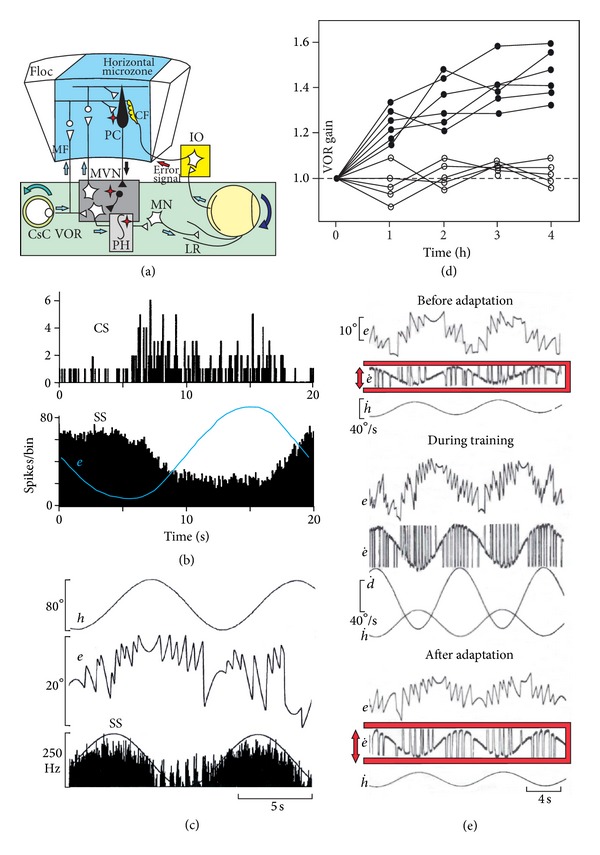
VOR adaptation and Purkinje cell behavior.  (a) Schematic diagram of the circuits connecting the VOR pathway including the medial vestibular nucleus (MVN) and the neural integrator located in the prepositus hypoglossa nucleus (PH) and the horizontal microzone of the flocculus (Floc). The error signal initiated by the retinal slip is conveyed via the inferior olive (IO) by the climbing fiber (CF). The asterisk points to possible sites of VOR plasticity (see text for details). (b) Peristimulus time histograms of the complex spike (CS) and the simple spike (SS) responses of a representative Purkinje cell in the horizontal zone of the rabbit flocculus to sinusoidal rotation at 0.05 Hz in the light. Note the reciprocity of the CS and SS firing (*adapted from* *Figure  2 of *[80] *with permission*). (c) Behavior of head velocity plus eye position sensitivity (HVplusP-P cell) recorded in the horizontal zone of the cat (*adapted from Figure  3 of *[24] *with permission*). (d) Time course of VOR adaptation corresponding to an out-of-phase VOR-OKN stimulation in 5 cats before (closed circles) and after (open circles) brainstem commissural incision. (e) Example of the VOR adaptation procedure used for increasing the VOR gain. Before the adaptation, the VOR is measured in the dark during table rotation (*h*) of 40°peak-to-peak at 0.10 Hz; the position of the eye (*e*) is recorded by means of the search coil technique. The VOR gain is the ratio between the peak-to-peak of the slow phase of the eye velocity and the peak-to-peak of the head velocity. During the training a random pattern of light circles was projected on the drum (d) surrounding the cat and oscillated out of phase of the head rotation, inducing an increase in the amplitude of the eye movements. After 4 hours of such training, the VOR was recorded in the dark and the VOR normalized gain increased by a mean gain of 1.41 ± 0.08 (*adapted from Figures  1  and  2 of *[77] *with permission*). Abbreviations: CsC, caudal semicircular canal; Floc, flocculus; Mn, motoneuron; OKN, optokinetic; LR, lateral rectus; VOR, vestibuloocular reflex.

The VOR stabilizes retinal images during self-induced and artificially induced rotational head movement by generating smooth eye movements that are opposite in direction and nearly equal in amplitude to head movement. When the compensatory eye movement is not adequately adjusted a retinal-slip error signal is generated (Figure 2). These errors are signaled by the CF input to the cerebellum. These inputs indexed by the occurrence of CSs cause LTD at the PF-PC synapses for the PFs that were active at or just before the arrival of the CF excitation. In order to contribute to adaptation of a reflex, a neural structure must receive information about (1) the sensory input which initiated the reflex, (2) the copy of the efferent commands, and (3) the resulting behavior.

Since the pioneering recordings of the PC in the floccular region in the behaving monkey by Lisberger and Fuchs [18, 68], the study of the horizontal zone of the flocculus in the cat and monkey has permitted to better understand the function of a cerebellar-microzone in the VOR adaptation in the horizontal plane of head rotation. First, the effectiveness of the flocculus in this process is supported by the fact that the electrical stimulation of this part of the cerebellum produces a smooth ipsilateral movement of the eye [23, 68, 69]. By such electrical stimulation, it has been possible to identify by antidromic invasion and collision technique the brainstem neurons projecting to the cerebellum. The cerebellar input signals of the median vestibular, prepositus and incertus nuclei were thus identified [22, 23, 25]. Pure head velocity and eye movement neurons (velocity plus position signals of the eye, so-called burst-tonic neurons) were recruited in the median vestibular nucleus [23], while burst neurons (eye-velocity) were identified in the prepositus and burst-tonic neurons in the prepositus [25] and incertus nuclei [22]. These inputs, thus, inform the flocculus about head triggering movement and the efferent copy of eye movement. The resulting behavior is given by the retinal-image slip signal relayed via the accessory optic tract to the direction-selective cells of the contralateral nucleus of the optic tract [70, 71], from which visual signals travel via the central tegmental tract to the dorsal cap of the inferior olive (IO). The IO is considered a crucial pathway in VOR adaptation [72–74] and also in the motor plasticity that compensates for vestibular damages [75]. As the majority of the mossy fibers (MFs) reaching the horizontal zone of the flocculus originated in the contralateral vestibular [23], the prepositus [25], the incertus nuclei [22], and the paramedian tract region [76], the section of the vestibular commissure in the cat resulted in the absence of VOR adaptation [77], and that in spite of that fact, the right and left flocculus were preserved. This demonstrates that VOR adaptation by the cerebellum necessitated the preservation of the contralateral MF inputs, contrasting with the classical wiring diagram of the VOR adaptation where only the ipsilateral vestibular input was represented. Diversity of firing related to eye-movement (acceleration, velocity, and position) was also recently reported in the brainstem neurons directly inhibited by the cerebellum [78]. These data corroborate the recent model of a feed-forward and feedback integrator [79] and shed a new light on the existence of neuronal plasticity inside the brainstem and cerebellum integrator network.

After the determination of the different inputs reaching a microzone, the output signals elaborated by the PC must still be identified in order to discover the input-output transformation realized by the cerebellar cortex. This approach is complicated by the marked complexity of PC behaviors of the same microzone compared to input signals, despite similarity of the various MF input signals conveying head velocity and eye velocity-position signals [24, 80] (Figure 2). Although sensitivity of PC to eye velocity prevails over other PC sensitivities [81–83], PC firing rate is far from being a mere reflection of a simple summation or combination of its inputs (with the notable exception of pure position PC, which can emerge from straightforward mathematical integration performed on the basis of the eye velocity input signals). For example, some PCs increase their firing when the eyes approach the central position of the gaze and linearly decrease their firing in both sides [24]. In this context, the definition of the adaptive parameters or the adoption of bioengineering function such as in the case of a neural integrator [84, 85] remains an arduous task. The fact that different sets of coefficients for eye position, velocity, and acceleration must be used to represent the SS firing during different visual-vestibular paradigm is not in accordance with a pure inverse dynamic model of the oculomotor plan, which should remain invariant across the different behaviors [86]. It is the reason why numerous signals such as head, retinal slip, and efference copy, have been linearly summed in order to model the PC during the VOR [87, 88]. Another difficulty arises from the observation that *in vitro* or in anesthetized preparation about 50% of PCs present three-mode state of firing (tonic, bursting, and quiescent) [89–91], switching from depolarized state (up-state), where simple spikes fire spontaneously, to hyperpolarized state (down-state), where only the complex spikes are present. This multimode of PC firing was also present in awake animals [44, 92–94] and is also complicated by the emergence of episodes of 600 Hz firing (so-called 600 Hz-buzz up-state) in about 15% of PCs in alert mice [95]. 

**Figure 3:**
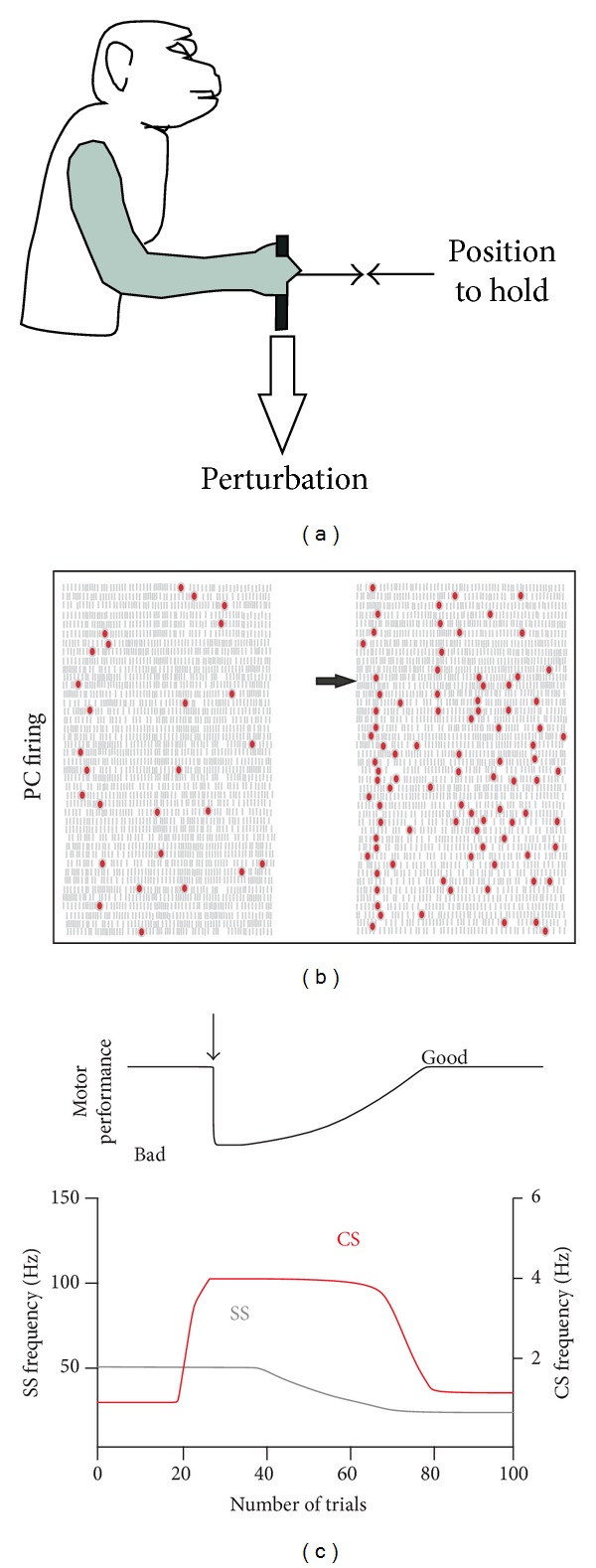
Gilbert and Thach (1977) experiment in monkey.  (a) Task consist to control a handle horizontally by flexing or extending the wrist to a central position and to hold it there despite flexor and extensor loads applied to the handle. (b) CS and SS frequencies change for a PC after a change in load (horizontal arrow). Each grey bar represents an SS and the red dots a CS. Each row of bars represents the discharge during a trial successively represented from top to bottom. Each flexor trace on the left, for which the monkey performance was good, is followed by an extensor trace on the right. At the arrow, the known extensor load of 300 g was modified to a novel 450 g inducing a strong decrease in the performance. (c) Idealized representation of the SS and CS firing a long time and the number of trials before load perturbation (vertical arrow), during the transition period from bad to good performance, and after. (*Adapted from* [36, 98] *with permission*).

Lisberger [96] proposed to extend the cerebellar learning theory to cerebellar internal models in which the LTD at the PF-PC synapses corrects the internal model stored in the cerebellum so that the next instance of a given movement became closer to perfection. According to Medina and Lisberger [83], three main hypotheses in the framework of the cerebellar motor learning theory need to be tested in the same awake animal preparation, namely, (1) when a movement is inaccurate the CF inputs are activated, (2) this input triggers synaptic plasticity and modifies the SS responses of the PC, and (3) these firing changes in the cerebellar cortex participate in the final adaptation of the motor behavior. The first study that paved the way for such an experimental view can be traced back to the pioneering work of Gilbert and Thach [36], demonstrating a clear increase of the CF inputs when the task performance (maintain a stable arm position in face of a mechanical perturbation) was inadequate, leading to a huge increase in the CS frequency until the performance was close to perfection (Figure 3). During this learning, SS firing progressively decreased to a stable state of lower frequency, considered as resulting from LTD. This first evidence of a dialogue functionally linking CS and SS during motor learning was confirmed by Ojakangas and Ebner [97].

**Figure 4:**
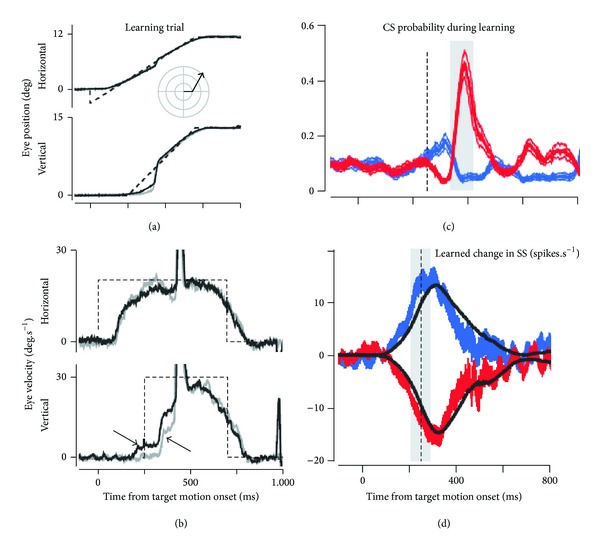
Directional learning during pursuit eye movements. (a) Representative eye movement during pursuit of a target moving first toward the right and then in oblique upward direction, the inset indicates the target motion in polar coordinates. (b) Eye velocity trace of the movement illustrated in (a). Gray and black traces indicate data from representative trials before learning and after at least 100 learning trials, respectively. The dashed traces indicate the velocity step signal of the target motion. Note that the vertical step (lower part) delayed the horizontal one (upper part) by 250 ms. The arrow pointing down and right indicates the learned response. The arrow pointing up and left indicates the hardwired visual response to the change in target direction, (*adapted from *[83]* with permission*).

By adapting this motor perturbation paradigm to the eye pursuit system, Medina and Lisberger [83] were the first to study trial-over-trial the neural changes occurring in the PC of behaving monkeys (Figure 4). In this original paradigm, the learning task consisted of tracking a visual target moving horizontally (toward to right), and then suddenly shifting to an oblique direction following an “instructive” stimulus in the form of a vertical (upward) velocity step occurring 250 ms after the horizontal one. The presence of a learned behavior was indexed by the occurrence of an upward movement of the eye before the onset of the upward movement of the target. For the majority of PCs, the learned change in SS firing (as indicated in Figure 4) showed the same shape and direction as the changes in eye velocity. Moreover, this PC spiking preceded the learned velocity component of the eye movement by about 30 ms, reinforcing the clear cause-effect relationship between the SS firing and the learned movement [99–102]. The situation of the CS firing in this process is more complicated because the instructed cellular changes related to the CF input produce their effect on the SS firing of the subsequent trial [83]. Moreover, these authors also reported that a learned SS response could occur when the CS firing remained at baseline levels and thus could not signal “errors.” They concluded that these data strongly support the cerebellar learning theory, although the CS-instructed PC plasticity is not the only mechanism for pursuit learning. This conclusion does not exclude other cellular mechanisms and other sites of plasticity in the different neuronal structures implicated in the final behavior.

As different levels of plasticity are possibly involved in the final operation exerted by the cerebellar cortex, it is difficult to identify the contribution of each level. As regards the granular layer, all GCs are required for the acquisition of new memories, whereas only a minority of GC is sufficient for the maintenance of basic motor performance [103–105]. Yet, the question about the contribution of the granular layer to the final learning processing remains open. Nevertheless, many slice studies converge to support a bidirectional NMDA-receptor-dependent plasticity at the synapse between MF and GCs [106–112]. Recently, Huang et al. [113] elegantly demonstrated that a same GC receives convergent input relaying proprioceptive sensory information coming from the external cuneate nucleus, and efferent motor copies coming from the cortex via the pontine nucleus. This sensory-motor convergence onto GC confirms the prediction of Marr [114] and Albus [115] about the associative faculty of this neuronal population, which is the most numerous in the central nervous system. These convergent entities are not uniformly distributed, but are organized in hotspots that are functionally linked to a same part of the body [113]. Before this important discovery, the multimodal nature of MF input [22, 23, 25, 116, 117] was interpreted as suggesting that the sensori-motor integration of these signals was realized at the level of the PC, where learning was encoded as the strength of the PF-PC synapse. On the contrary, the consequence of GC convergence is that the input of the PC already contains prefabricated signaling about efferent motor copies and proprioceptive state. In line with hierarchical network organizations, Huang et al. [113] propose that this GC sensory-motor integration may allow the PC to accomplish more complex learning tasks. Another view endorsed by Hatten and Lisberger [118] is that this early sensori-motor convergence at the GC may indicate a limited ability of the cerebellum to adjust the gains of the sensory and motor signals in an independent way. This stresses the importance of plasticity at the level of the MF-GC synapses. 

## 4. Plasticity in the Eyeblink Conditioning Reflex: The Second Cerebellum Lesson

Classical eyeblink conditioning constitutes one of the most used experimental models for investigating the neural mechanisms underlying motor learning. The conditioning of eyelid/nictitating membrane response was first studied in humans ([119, 120], for a review) and popularized later in animals by Gormezano's group along 60's [121, 122]. Animal findings are of particular importance because both cerebellar lesion and functional brain imaging data obtained in humans are in good agreement with those coming from animal models [123, 124].

**Figure 5:**
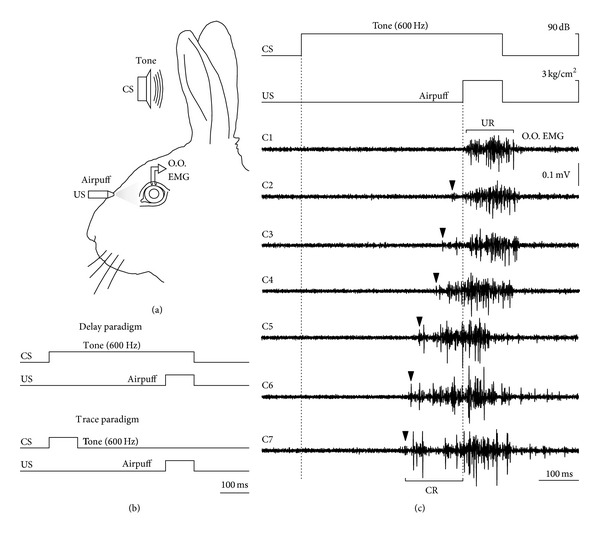
Eyeblink  conditioning  protocols  and  electrophysiological  recording  of  the  conditioned  eyelid  response. (a) Experimental design for eyeblink conditioning in behaving rabbits illustrating the location of the bipolar hook electrodes for the recording of orbicularis oculi muscle activity (O.O. EMG). Classical eyeblink conditioning protocol consists of pairing a conditioned stimulus (CSt) (e.g., a neutral stimulus such as a tone) and an unconditioned stimulus (USt) (e.g., an airpuff to the eye that induces a reflexive blink). (b) Two principal paradigms have been classically used depending on the temporal relationship between CSt and USt. Thus, in the delay paradigm (top) the CSt and USt coterminate. In the trace paradigm there is a constant time interval between both stimuli (bottom). (c) The figure illustrates the eyeblink conditioning process using a delay paradigm. The conditioning paradigm (CS and US presentations) and representative orbicularis oculi electromyohraphic (O.O. EMG) recordings from the same animals along seven conditioning sessions (C1–C7) are presented. Along conditioning sessions the initial unconditioned response (UR), consisting of a reflexive eyelid response just after the US, leads to a timed eyelid response which precedes the USt named the conditioned response, CR (arrows).

Classical eyeblink conditioning protocol consists of pairing a conditioned stimulus (CSt) (e.g., a neutral stimulus such as a tone) and an unconditioned stimulus (USt) (e.g., an airpuff to the eye that induces a reflexive blink) (Figure 5(a)). Two principal paradigms have been classically used depending on the temporal relationship between CSt and USt. Thus, in the delay paradigm the CSt and USt coterminate, whereas in the trace paradigm there is a constant time interval between both stimuli (Figure 5(b)). Along conditioning sessions, the initial unconditioned response (UR), consisting of a reflexive eyelid response just after the USt, leads to a timed eyeblink response which precedes the USt (named the conditioned response, CR) (Figure 5(c)). It has been proposed that each one of these paradigms can engage different neuronal circuitry, in such a way that only brainstem and cerebellum are required for delay conditioning [125], and hippocampus and other forebrain structures are required for the more cognitively complex trace conditioning [126, 127], where the CR occurs in the absence of any sensory stimulus. Nevertheless, taken into account that sensory receptors are activated by changes in the presented stimulus (and not by its sustained presence), some authors considered the delay conditioning as a particular case of trace conditioning [124].

One of the factors that have contributed to considering eyeblink conditioning as one of the most important experimental models for cerebellar motor learning studies partially relies on the knowledge of the anatomical and functional pathways conveying CSt and USt information to the cerebellum [128, for a review]. Focusing on the cortical cerebellar level, CSt information reaches PC trough PFs, whereas US information is conveyed by CFs. As the PC axons are the sole output of the cerebellar cortex, the convergence of both stimuli on the PC seems to be crucial for new motor skills learning. This idea was first proposed by Marr [114], who associated CF inputs with an “error” signal able to modify the contextual information coming from PF-PC synapse. According to the inhibitory nature of PC output, Albus proposed [115] that the conjunction of these two signals could induce a decrease in the PF-PC synaptic strength (long-term depression, LTD), leading to a disinhibition of the deep cerebellar nuclei (DCN), giving rise to a CR. Beyond the understanding of the pathways and their physiological meaning, eyeblink conditioning allows for simultaneous neural recording, lesion of the neural tissue, and local pharmacological manipulation of the implicated areas in the behaving animal [129]. In addition, the learning process can be properly indexed by an accurate recording of eye and eyelid movements or eyelid associated muscle activity [130].

During the last decade, the cerebellum has been considered the principal structure associated to eyeblink conditioning containing, together with the brainstem, the entire essential circuitry involved in classical conditioning [128]. According to this brainstem-cerebellar model, the pontine nucleus receive projections from both cortical and subcortical structures implicated in conveying CSt information (auditory, somatosensory, visual and association systems) [131–134] and sending axons (MFs) directly to the cerebellar cortex and the DCN, particularly to the interpositus nucleus (IN) [135–137]. On the other hand, USt information is conveyed by somatosensory inputs relaying in cranial nuclei and projecting to dorsal accessory olive. Neurons in the dorsal accessory olive send CF projections to the cerebellar cortex giving collaterals to IN [138–140]. According to this circuitry, there are two major cerebellar structures where CSt and USt signals seem to convey the IN and the cerebellar cortex at PC layer level.

Several evidences point to the importance of the IN in classical eyeblink conditioning. Thus, it has been early demonstrated that ablation of the lateral cerebellum and the electrolytic lesions of the dentate and interpositus cerebellar nuclei caused a near-complete abolition of the CR with no effect on UR [141, 142]. In addition, pharmacological manipulation inactivating IN by using the local anesthetic lidocaine [143] and the GABA_A_ receptor agonist muscimol [144] in naive animals prevented eyeblink conditioning. Nevertheless, local injections of muscimol in the IN in half-conditioned animals decreased the amplitude of CRs with no effect on the percentage of CRs, suggesting a major role of the IN in the performance of eyelid responses rather than in the learning process [145]. Electrophysiological recording of neuronal activity in the IN during eyeblink conditioning shows a strong correlation with the conditioned eyeblink response [2, 146–149]. Nevertheless, there are important discrepancies about the precise latency of the IN neuronal discharge in relation to the CR onset. Whereas some authors report IN activity from discharges prior to the execution of the learned eyeblink response [146, 147], other authors show that identified cerebellar IN neurons start firing after the beginning of the CR when eyelid movement is accurately recorded by using magnetic search coil technique [2, 148, 149]. These discrepancies have led some authors to propose the participation of this nucleus in the timing and performance of ongoing CRs rather than the generation and initiation of the CRs [124, 150–152].

As mentioned before, the second cerebellar structure, where CSt and USt signals seem to convey, is the cerebellar cortex. PC constitutes the only output of the cerebellar cortex, modulating through its inhibitory synapse the activity of DCN [153]. Although the CFs make excitatory synapses in the DCN, they mainly project to PCs in such a way that each PC receives input from only one climbing fiber [154]. Major experimental evidence for the implication of the cerebellar cortex in eyeblink conditioning comes from selective lesion experiments [155–159], electrophysiological unitary recording [160–162], pharmacological manipulations [145, 163–165] and loss of PC type in mutant and knock out mice [166–170]. Regarding the selective lesion of the cerebellar cortex, two main regions have been involved in CR production and timing, Larsell's lobule HVI [155–157] and the cerebellar anterior lobe (lobules I-V) [158, 159]. Interestingly, the effects of lobule HVI lesion consisted of an initial loss of CRs percentage with partial relearning with retraining [157], whereas the lesion of the anterior lobe shortened the onset and peak latency of CRs with no change in CR percentage [158], suggesting that both cerebellar regions are implicated in different aspects of conditioning. The recording of PC activity in these two regions shows patterns of activity (excitatory and inhibitory) that seem to be related with the CR execution, in addition to CSt and USt presentation [160–162]. In PC, prenatally exposed to ethanol (fetal alcohol syndrome, FAS mice), presenting decreased voltage-gated calcium currents because of a decreased expression of the *γ*-isoform of protein kinase C, the PF-PC LTD was converted into long-term potentiation (LTP) [47]. This alteration was accompanied by a deficit in eyelid conditioning in the early phase of the learning (day 1 and 2), but FAS mice finally reach a same score as wild type mice (80% of CR) at day 5 (Figures  1(e) and  1(f) of [47]), demonstrating that absence of LTD in slice preparation does not compromise the final acquisition of eyelid conditioning but only invalided the early phase of the process.

Because DCN constitutes the only target for cerebellar cortex output, the pharmacological disconnection of these two structures seems to be an important approach for understanding their respective roles [171]. In particular, because of the GABAergic nature of synaptic transmission between PC and DCN, the use of GABA_A_ receptor antagonist gabazine and GABA_A_ chloride channel blocker picrotoxin has been explored [163–165]. Picrotoxin and gabazine infusions disrupt the timing and the amplitude of the CR [163, 164]. In addition, the effects induced by the injection of GABA agonist muscimol differ from the abolishment of the CRs [163] to a decrease in the amplitude of the CR [145], respectively. Finally, one of the most convincing demonstrations of the cerebellar cortex role in eyeblink conditioning comes from the use of mutant mice with PC degeneration [168, 169] and gene knock out mice [166, 167, 170]. Mutant mice with PC degeneration exhibited a significant reduction in the CR percentage and shorter peak latencies in comparison to CRs in wild-type mice [168]. This reduction in eyeblink conditioning has been confirmed in knock out mice with specific inactivation of metabotropic glutamate receptor type 1 (mGlu1) gene or glial fibrillary acid protein (GFAP) gene; in the latter, where impairment of PF-PC synapse LTD has also been reported [166, 167, 170]. Putting together all this evidence it is clear that the cerebellar cortex plays an important role in eyeblink conditioning. Nevertheless, experimental results evidenced that animals may learn “slowly” without the cerebellar cortex suggesting that the relative contribution of the cerebellar cortex and deep nuclei depend on the amount and type of training [153].

As commented before, the cerebellum and its relation with the pontine nuclei have classically constituted the focus of attention in eyeblink conditioning studies even when a large number of brain regions are implicated in the performance of eyeblink [124]. Thus, the injection of retrograde trans-synaptic tracers (attenuated rabies virus) in the orbicularis oculi muscle of the rat [172] shows that beyond brainstem and cerebellum, other brain regions like the cerebral cortex, the limbic system, or visual related structures are related with this apparently simple motor system. Due to its functional and anatomical relation with eyeblink, two different structures are potentially relevant for eyeblink conditioning, that is, the motor cortex and the red nucleus. Regarding the motor cortex, although early lesion studies reported contradictory results [173, 174], different studies suggest a revision of its possible implication in eyeblink conditioning. Thus, recent anatomical results demonstrate the existence of a monosynaptic pathway from vibrissa motor cortex to facial motor neurons in the rat [175]. Moreover, electrophyisiological recordings show the activation of neurons in the motor cortex preceding the onset of CR [176, 177], and interestingly enough, recent results coming from local reversible inactivation of the motor cortex [178] point to an underestimated role of this neuronal region in eyeblink conditioning. Otherwise, the red nucleus, that receives inputs from sensoriomotor cortex and cerebellar nuclei and projects to facial and accessory abducens nuclei [179], has been classically considered as a mere relay center [171]. Nevertheless, some studies propose a more active role of red nucleus in motor learning processes [178, 180, 181], particularly when afferent inputs, as the motor cortex, are transiently removed during the acquisition process [178]. Previous evidences on motor cortex and red nucleus support the distributed idea of motor learning leading some authors to consider that the cerebellum is involved in the performance of CR more than in its acquisition [124, 150–152].

## 5. Plasticity of the Cutaneous “Envelope” Representation: The Third Cerebellum Lesson

Comprehensive sensory information from the skin collected from the numerous specific types of sensors can be represented as a cutaneous envelope, which is the physical site of interaction between the body and the environment. This envelope is well represented in the cerebellar mantle, where sensory information conveyed both by the CF and MF is integrated. There is a functional relationship between this sensory envelope and the proprioceptive afferents from the underlying muscles. In turn, it has been hypothesized that the cutaneous envelope is functionally linked to the control of the related joint movements [182], and this may constitute another field of action for cerebellar plasticity.

As in the case of the control of eye position by the flocculus described above, elemental movements allowing the optimization of joint position are assumed by distinct cerebellar areas. One of the most studied circuits concerns the forelimb movement control in the cat. The functional organization of cerebellar regions contributing to this control has been studied with regard to input-output anatomical connections. Each cerebellar module has thus been shown to receive CF afferents from a specific IO region and projects the PC axons to a specific part of the DCN. Each cerebellar module [183] can be further divided into different microzones, considered as the operational units of the cerebellum [182, 184, 185]. The existence of microzones is reinforced by the presence of regular patterns of mediolaterally disposed bundles of medium-sized myelinated PC axons separated by darker staining slits of smaller fibers [186]. The microzone organization assumes a functional convergence between a CF cutaneous receptive field and a specific movement to be controlled. For example, the C1/C3 zones of the anterior lobe in the cat present 30 to 40 microzones that project to the nucleus interpositus and control movements via the rubrospinal and corticospinal tracts [182, 187]. The information transmitted by the CF is multimodal and originates from cutaneous A beta (tactile), A delta, and C (nociceptive) fibers, and from muscle afferents [188]. 

The cutaneous receptive fields of the CF projecting onto the C3 zone possess a detailed spatial organization divided into eight functional classes [189]. The cutaneous and proprioceptive modalities are transmitted by single CF [190]. Although the stimulation of the muscle afferent would tend to move the cutaneous receptive field of the CF toward the skin stimulus, the movement initiated by the cerebellar module tends to move the cutaneous field away from the stimulus. This is interpreted as a braking action performed in order to escape the stimulus considered as an “error” signaling transmitted by the CF [182]. 

Cerebellar LTD was first demonstrated in response to conjunctive stimulation of the vestibular nerve and the IO in decerebrated rabbit by Ito et al. [191], and in response to conjunctive PF and IO stimulation by Ito and Kano [192]. Subsequently, Ekerot and Kano [193] reproduced the same LTD effect in the anesthetized and decerebrated rat. Later, the same authors specified that the maximal effectiveness for inducing LTD was obtained for a time interval between the CF and the PF stimulation of 125 to 250 ms, and that the conjunctive stimulation given at 4 Hz induced a stronger LTD than those obtained at 1-2 Hz [194]. 

The same plasticity paradigm was reactivated more recently by Jörntell and Ekerot [195] and enriched by the outstanding experience that this group had acquired on the receptive fields of the CF and MF integrated in the C3 zone of the anterior lobe [196]. These experiments were realized in anesthetized and decerebrated cats. The cutaneous receptive field of the CF and PF inputs to the PC and molecular interneurons was first identified by tactile stimulation of the skin over several body areas. Then, the authors used 5 minutes of burst microstimulations (15 pulses at 100 Hz repeated every third second), paired or unpaired, with CF activity applied on the superficial layer of the cerebellar cortex in order to induce plasticity of PF input. 

The main result was that unpaired burst stimulation of the PF beam durably transformed (~2 hours) the original restricted SS receptive field to a wide receptive field resembling the extended field described for the MF input [197]. In contrast, when the same stimulation was applied on the PF 2–5 ms after the spontaneous occurrence of a CS, the receptive field of the PC was strongly and durably (~1 hour) reduced, and the peripheral excitation of the PC was abolished for about 5 minutes. As this plasticity paradigm uses mixed natural (peripheral skin touch) stimulation and artificial cerebellar microstimulation, it is important to note that the same extension of the receptive field was reproduced by using only electrical stimulation of the skin area (333 Hz trains lasting 150 ms, repeated at 1 Hz for 10 min) [195]. In contrast, when the PF burst was triggered by a spontaneous CS, an opposite effect occurred, that is, restriction of the field. Moreover, this conjunctive stimulation produced a depression of the excitatory input and an increase of the inhibitory input of the PC and an increased activity of the interneurons [195]. The most dramatic but complex phenomenon in these experiments is receptive field plasticity. Different hypotheses have been advanced by these authors to explain the gating mechanism induced by electrical stimulation of the cerebellar surface. In this context, we here propose to consider the PC firing behavior and its peripheral SS receptive field as a somatotopic representation of the body skin envelope. From the input standpoint, the expansion or reduction of the peripheral territory would then mean that the peripheral cutaneous receptors that are potentially active for providing sensory signal to the MFs occupy a territory that goes well beyond the actual representation of the PC [197]. This is consistent with Jörntell's and Ekerot's [195] findings, that would thus suggest that PF beam burst stimulation is able to induce plastic change of this representation by changing the SS firing of the PC and those of the molecular interneurons in a reciprocal way, the sign of the modification depending on the presence of CF input. Jörntell's and Ekerot's (Figure  7 of this paper) [195] thus suggested that gating modulation may occur at the level of the PF-PC synapses. The cutaneous receptors situated outside the PC receptive field but which anatomically project on PC via the GC are not able to activate the PF-PC synapse before the burst stimulation of the PF beam but are potentiated after the stimulation. As the Golgi cell can receive an excitatory input from the burst stimulation of the PF beam, it could therefore exert plasticity at the level of the granular layer. In addition, the modified PC output could also be actively involved in the control of the MF-GC synapse via increased inhibition of the Golgi cell, which can in turn facilitate the activation of the GC, increasing *in fine* the PC receptive field. A reversed effect could be produced at the same level when an LTD is induced at the PC reducing its inhibition on the Golgi cell, which can in turn inhibit the GC decreasing the PC receptive field.

**Figure 6:**
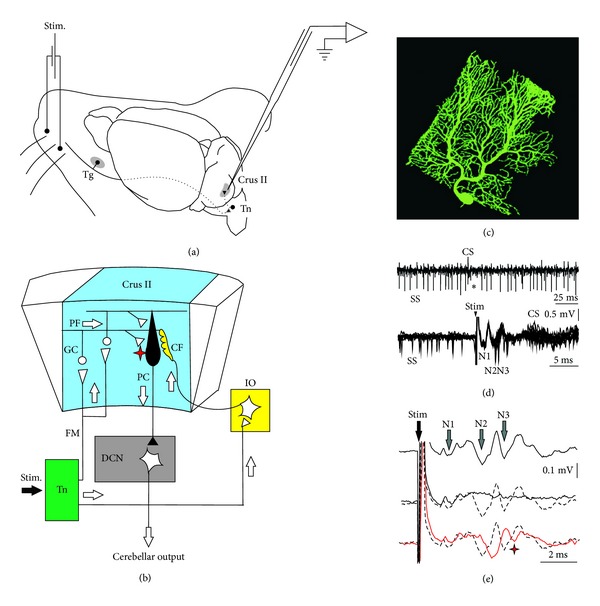
Experimental design and electrophysiological response to electrical stimulation of mouse whiskers. (a) Animals were prepared for chronic recordings of local field potentials (LFP) and unitary extracellular activity in the Purkinje cell layer of the Crus I/II area. Facial dermatomes of the whisker region were electrically or tactilely stimulated with a pair of needles under the skin (Stim.) pulse, respectively. Sensory information comes into the Crus I/II area from the trigeminal nucleus (Tn) in the brainstem, which receives afferent signals from the trigeminal ganglion (Tg). (b) Schematic diagram of the circuits linking the trigeminal input and the olivo cerebellar system. (c) Photography of a single PC injected by Lucifer yellow. (d) Recording of spontaneous firing behavior of a Purkinje cell (PC) shows the presence of single spikes (SS) and complex spikes (CS). The presence of a CS followed by a pause in the SS firing (asterisk) identifies this neuron as a PC. Single trials, superimposed (*n* = 11), show spontaneous firing before the whisker electrical stimulation (Stim) and the temporal reorganization of the firing after the stimulus. SS firing occurred at the low points of the N2 and N3 components and later. The evoked CS occurred at a latency of 9–13 ms after the stimulus onset (arrowhead). (e) Long-term depression on the LFP is evident after the 8 Hz stimulation protocol (red line), when the latency of the N2 components increased and the amplitude of the N3 component strongly decreased. These effects were maximal just after the 8 Hz stimulation and persisted for at least 30 min. Single traces selected to compare latencies and amplitudes of LFP components; (top) before 8 Hz stimulation protocol, (middle) during 8 Hz stimulation, and (bottom) just after 8 Hz stimulation protocol. The red asterisk indicates the LTD effect on N3 postsynaptic components after 8 Hz stimulation, (*adapted from *[37]* with permission*).

In line with the functional proposal of the Swedish group, the possibility that the PC output could modulate the muscle tension (via the IN, the red nucleus, and the motor cortex) and the proprioceptive input to the MFs also needs to be revisited. Two main points of this experimental approach need to be discussed: (1) the unphysiological character of the PF stimulation paradigm and (2) the decerebrate preparation. Concerning the first point, Jörntell and Ekerot [198] have introduced a new plasticity paradigm, where the skin itself was stimulated with electrical burst (333 Hz for 150 ms) delivered at 1 Hz for 5 min. These stimulations were paired or not with IO stimulation and reproduced the same type of receptive field plasticity: increasing of the PC receptive field (skin-LTP protocol) when the 333 Hz bursts were applied alone on the skin and decreasing of the PC receptive field (skin-LTD protocol) when 333 Hz bursts were associated to the IO stimulation [198]. When the whisker pad is naturally stimulated by airpuff [199, 200] or by electrical pulses delivered in the control situation at random rate (~0.1 Hz) or at 8 Hz (for inducing LTD) [37], the PC of the Crus II zone presents a highly reproducible firing comprising an early SS response shortly followed by a CS (Figure 6). This indicates that a single skin area of the face conveys significant signal to both the MF (via the trigeminal nucleus) and CF (via IO). Moreover, after selecting the appropriate frequency rate for producing a significant effect on the N3 local field potential (LFP) of the PC layer, it was demonstrated that the electrical stimulation of the whisker pad at 8 Hz during 10 minutes induced an LTD effect at the PF-PC synapses [37]. While the effect of this plasticity recorded in alert mice was not tested on the receptive field configuration plasticity as it was done in Jörntell and Ekerot's work [198], it would be interesting to know if this PC-LTD is also evoked by 8 Hz stimulation when applied on the skin over the rest of the body.

Still, the recent studies of our group [37, 201] showing that N2 and N3 LFP components related to the PC spiking are delayed during at least 30 minutes after 8 Hz conditioning stimulation of the whiskers pad reinforce the timing hypothesis and the existence of a specific mechanism of plasticity acting on the time constraint. Interestingly, this timing plasticity is absent in all the mouse presenting a PC-specific ablation of the large-conductance voltage- and Ca^2+^-activated K^+^ (BK) channels [201], while the decrease in N3 amplitude is inconsistent. This indicates the implication of BK channels in the timing plasticity of the PC. This is in accordance with the crucial role played by these channels on the rhythmic imprint of the PC firing [199] and on the final control of movement [202]. This timing plasticity is conserved in the mdx mouse model of Duchenne muscular dystrophy, while the amplitude depression (LTD) of the N3 component is absent (C. Prigogine, J. Márquez-Ruiz, B. Dan, and G. Cheron, personal communication). Interestingly, the PC of the mdx mice is concerned with the deletion of the dystrophin gene, resulting in disorganization of the GABA_A_ receptors stabilization and clustering at postsynaptic densities of their inhibitory synapses [203]. This clustering disruption impairs the function of these inhibitory synapses and leads to a decreased inhibitory input of the PC and increased SS firing rate in alert mdx mice [204]. The absence of LTD on N3 amplitude may be explained by imbalance in PC excitatory-inhibitory input altering the LTD process but conserving N2-N3 timing plasticity. Experimental data from the two latter mouse models (BK-cer and mdx) show that the two types of plastic changes of the LFP, namely, N2-N3 time shift and N3 amplitude decrease, involve different mechanisms.

Concerning the use of decerebrate preparation, the recent data of Huang et al., [113] demonstrating the important contribution of cortico-pontine information intricately mixed with peripheral information at the level of the single GC, call in favor of the preservation of these cerebral input when studying cerebellar plasticity. Moreover, the presence of long-term plasticity in the mouse sensorimotor cortex induced by passive whisker stimulation at 8 Hz shows that the cortico-pontine input may transmit direct or learned signals to the cerebellum [205]. The imbrication of cerebral and cerebellar plasticity loops can be viewed as a new challenge when deciphering the physiological mechanism of sensori-motor gating and learning. 

The next step will be to fill the gap between animal and human studies about gating and learning of the central representation of the cutaneous envelope. For example, in human the N30 component of the somatosensory evoked potential following electrical stimulation of the median nerve can be suppressed when the stimulated hand is moving [206, 207] or when the subjects only imagine moving their fingers [208]. Based on the above, it will now be important to study the involvement of the cerebral cortex and the cerebellum and bidirectional communicating loops in both situations. This approach should be extended to other human plasticity paradigms, such as the learning effect of 10 minutes of finger movement repetition [209] or action observation [210] on the directional tuning of movement induced by transcranial magnetic stimulation of the cerebral cortex. In addition to the contribution of LTP in this cortical plasticity, [209, 211] the cerebellum is probably involved in this cerebral plasticity [212, 213]. As suggested by these latter authors, this could be accomplished by bidirectional spike-timing dependent plasticity through the cerebello-dentato-thalamo-cortical pathway and the primary motor cortex. As suggested by Censor et al., [214], similarities in temporal dynamics occurring in different parts of the brain could be a key element to the existence of a general mechanism for learning in humans. In this context, spike-timing dependent plasticity may occupy a central position in learning mechanism [215], by which the cerebellum will be on the pole position, at least for deciphering fundamental and translational mechanisms from cellular to network levels in behaving humans.

## Abbreviations

BK: Large-conductance voltage- and Ca^2+^-activated K^+^ channel
CF: Climbing fiber
CR: Conditioned response
CS: Complex spike
CSt: Conditioned stimulus
DCN: Deep cerebellar nuclei
EPSP: Excitatory postsynaptic potential
FAS: Fetal alcohol syndrome
GABA_A_: *γ*-aminobutyric acid receptor type A
GC: Granular cell
GFAP: Glial fibrillary acid protein
IN: Interpositus nucleus
IO: Inferior olive
IPSP: Inhibitory postsynaptic potential
LFP: Local field potential
LTD: Long-term depression
LTP: Long-term potentiation
MF: Mossy fiber
PC: Purkinje Cell
PF: Parallel fiber
SS: Simple spike
UR: Unconditioned response
USt: Unconditioned stimulus
VOR: Vestíbulo-ocular reflex.
